# Entitlement-Based Access Control for Smart Cities Using Blockchain †

**DOI:** 10.3390/s21165264

**Published:** 2021-08-04

**Authors:** Fariza Sabrina, Julian Jang-Jaccard

**Affiliations:** 1 School of Engineering and Technology, Central Queensland University, Sydney, NSW 2000, Australia; 2 Cyber Security Lab, School of Natural and Computational Sciences, Massey University, Auckland 0632, New Zealand; j.jang-jaccard@massey.ac.nz

**Keywords:** blockchain, smart contract, Internet of Things (IoT), smart city, access control, security

## Abstract

Smart cities use the Internet of Things (IoT) devices such as connected sensors, lights, and meters to collect and analyze data to improve infrastructure, public utilities, and services. However, the true potential of smart cities cannot be leveraged without addressing many security concerns. In particular, there is a significant challenge for provisioning a reliable access control solution to share IoT data among various users across organizations. We present a novel entitlement-based blockchain-enabled access control architecture that can be used for smart cities (and for any ap-plication domains that require large-scale IoT deployments). Our proposed entitlement-based access control model is flexible as it facilitates a resource owner to safely delegate access rights to any entities beyond the trust boundary of an organization. The detailed design and implementation on Ethereum blockchain along with a qualitative evaluation of the security and access control aspects of the proposed scheme are presented in the paper. The experimental results from private Ethereum test networks demonstrate that our proposal can be easily implemented with low latency. This validates that our proposal is applicable to use in the real world IoT environments.

## 1. Introduction

Internet of Things (IoT) today is making an enormous impact in our daily life by allowing smart devices and sensors connected to the Internet to provide valuable services. The smart city is one of the major IoT applications that utilize the recent advances in IoT providing low-cost computing and interconnectivity. A large IoT network within a smart city can connect a huge number of devices efficiently. However, the interconnectivity of large heterogeneous devices that are used for the IoT network to collect, process, and disseminate IoT data could pose serious security concerns [1,2,3,4].

One of the most significant security concerns that a large-scale IoT application such as smart cities faces today is provisioning an efficient and reliable Access Control (AC) scheme. The traditional AC approaches, such as the Role-Based Access Control (RBAC), the Attribute-Based Access Control (ABAC), and Capability-Based Access Control (CapBAC), are no longer able to provide a scalable, manageable, and efficient method for IoT environments [5,6,7,8]. This challenge can be magnified especially in large-scale IoT environments where the data is shared across multiple domains, including third parties. In this environment, AC schemes must ensure that the data owner is in full control of their own data while being able to safely delegate the necessary access rights to others [7,9].

There has been a growing interest among researchers in using blockchain technology for access control solutions in IoT [5,6,7,8,9,10,11,12,13,14,15,16]. We argue that there are several issues associated with the existing offerings:

- High latency: all access authorization regardless of the mode of operation (e.g., read, write) are considered as blockchain transactions that require computationally expensive mining operations before the client can access resources [17]. It not only adds significant latency but is not scalable in a large-scale IoT network [6,11].
- Lack of flexibility: no delegation support for transferring access rights beyond the trust boundary of an organization [5,14,16].

To address the limitations stated above, we present a novel entitlement-based access control architecture that is flexible, scalable, and supports low-latency for smart cities and any other large-scale IoT applications. The main contributions are summarized as follows:

1. We propose a novel entitlement-based blockchain-enabled access control architecture for a large-scale IoT environment where resources can be shared across multiple organizations. Our proposal provides a flexible access delegation and revocation mechanism that ensures the resource owner is in full control. The proposed access control model also allows a user to have a different set of access rights on the same resource depending on the user’s profile contexts.
2. We provide detailed design and implementation of the proposed scheme on the Ethereum blockchain platform. Extensive experiments are performed on a private Ethereum network with multiple instances of test client applications concurrently generating a high volume of blockchain transactions/calls and thus simulating a real-world scenario. Our feasibility study confirms that our proposal can be easily implemented using a publicly available blockchain.
3. We also present a qualitative analysis of how the proposed architecture can meet the standard security and access control aspects of a complex IoT environment.

The rest of the paper is organized as follows. Section 2 provides background and related work. Section 3 describes a smart city access control use case. Section 4 describes the proposed blockchain-enabled access control architecture. Entitlement Management and Access Control are described in Section 5 and the design of smart contracts on the Ethereum platform and algorithms are detailed in Section 6. Experimental set-ups and results are presented in Section 7. Section 8 presents the conclusion.

## 2. Background and Related Work

Internet of Things (IoT) can be described as a collection of connected devices and sensors that have been deployed to gather data from the given environment. In recent years, the advancement of IoT technologies has contributed towards the significant development of smart cities for sustainable living, reduction in energy wastage and costs, and increased comfort and productivity for citizens [18,19]. A smart city has many domains such as smart parking, smart hospitals, smart utility, smart transport, smart traffic authority, smart office buildings etc. Smart cities are a real driver for connecting application domains for IoT scenarios and need collaborative access to IoT resources data both within an organization and cross-organization scenarios.

A large IoT network within the smart city can connect a huge number of resource-constrained devices with various storage and computing capabilities and it demands low latency for data gathering and access. However, the heterogeneous nature of such an IoT environment within a smart city poses significant privacy and security concerns [18,19,20,21,22,23].

Many researchers have recently explored the challenges in IoT-based smart cities and have proposed potential applications of blockchain technology [20,21]. One of the key challenges of smart city is to devise a solution for protecting data and information and devising an access control mechanism where the different domains within smart cities can safely share the data without compromising control, confidentiality, and privacy [21].

Access Control (AC) is a fundamental method for any IT system aimed to prevent unauthorized access to organizations’ assets (e.g., data). In recent years, there have been many proposals of AC schemes to address the security and privacy challenges in IoT. Some of these proposed schemes are based on traditional AC schemes, such as RBAC (role-based access control), ABAC (attribute-based access control), and CapBAC (capability-based access control) while others utilize blockchain technologies to devise new AC models or to augment traditional ABAC or CapBAC models. The RBAC-based models [24,25] provide solutions where an access right (e.g., read, write, update etc.) is defined for specific resources based on the roles of the data owner within an organization unit. The model cannot be used in a complex environment such as IoT where an access right may need to be given to external organizations and third parties, as it is not feasible to map all potential roles to different resources and enforce associated security constraints.

The ABAC-based models [26,27,28] define security policies by associating various types of attributes to the subjects (users) and objects (resources). Ding et al. [8] presented an ABAC scheme for IoT that uses blockchain technology for the distribution of attributes. Wang et al. [10] proposed a distributed ABAC scheme using blockchain for IoT which uses multiple smart contracts for managing subject attributes, object attributes, and access controls. Islam and Madria [11] proposed another ABAC system for IoT that uses a permissioned blockchain and smart contracts. While ABAC models address some drawbacks of traditional RBAC approaches by providing more fine-grained access controls in some application scenarios, still they are not suitable for large-scale IoT environments since it is not feasible to define all attributes involved, corresponding security policies, and agreements.

In CapBAC-based models [29,30], a subject (user) is associated with a list of capabilities (access rights) required for accessing all concerning objects (resources). In this model, AC is provisioned by an unforgeable and transferable capability token which contains the explicit list of access rights and their associated access conditions and security constraints. Xu et al. [5] proposed a blockchain-enabled CapBAC scheme where a capability token is deployed in a public BC. However, these CapBAC-based models lack flexibility for how a granted access to a resource can be further delegated and shared within a large organization and cross organizations.

Zhang et al. [6] and Sultana et al. [12] proposed to use multiple smart contracts such as register contract, access control contract, and judge contract in blockchain to achieve distributed access control for IoT systems. Ouaddah et al. [13] proposed another blockchain-based access control framework for IoT which uses bitcoin-like transactions for provisioning and transferring access tokens. However, these proposed schemes cannot scale and have a high latency since the access authorization is fully dependent on blockchain transactions that are resource intensive and requires longer response times.

Alphandy et al. [14] proposed a security architecture for IoT that uses public blockchain for resource authorizations but did not cover any access right delegation aspects. Novo [15] proposed a blockchain-based scalable access management scheme for IoT but focused on permission management for IoT devices only. Zhaofeng et al. [16] proposed a blockchain- and smart contract-enabled decentralized trust management system focused on secure usage control of IoT Big Data but did not cover any aspects related to access rights delegation.

Lin et al. [31] proposed a blockchain-based data sharing scheme for smart cities where their main focus was to also prove that the proposed scheme can satisfy pseudonymity, traceability, efficient revocation, authenticity, confidentiality, and fairness of data sharing among multiple entities. The paper provided detailed models and some experimental results; however, their work did not include access control and access delegation aspects for smart cities IoT.

Saha et al. [32] proposed a blockchain-based access control protocol for IoT-enabled healthcare applications where a user can use his/her smart mobile device to connect to trusted hospital authorities that are within a private blockchain network. This work provides a theoretical analysis of the proposed solution, but no performance evaluation through experimental work has been provided.

Tapas et al. [33] presented some experimental studies with Ethereum-based smart contracts for access control and delegations in IoT. In this paper, the authors proposed an authorization and delegation model for IoT based on blockchain technology and which can be used for the management of nodes and resources. The authors provided some experimental results in terms of execution times on a private and test Ethereum networks for delegation creation and delete and for checking access requests. However, it is not clear whether the delegation model can be used for large-scale IoT scenarios with cross-organization access control needs.

Zhang et al. [34] proposed an attribute-based access control framework using blockchain smart contract for smart cities. They have used multiple smart contracts for managing ABAC policies, managing attributes of subjects and objects, and for controlling access. They have implemented the proposed scheme on a private Ethereum network and have measured the gas cost for the operations, but have not provided any results on the response time and throughput. This work also did not cover any access rights delegation aspects. The prototype has demonstrated a good performance in a small scale scenario and implementation of the work on large scale has been mentioned as future work.

In summary, existing approaches lack a flexible access control model with a robust access rights delegation capability that can be used in a single large organization as well as cross-organizational scenarios. These existing methods also do not support the scalability and performance needed for large-scale IoT environments. Our proposal aims to address these issues.

## 3. A Smart City Use Case

A smart city has many organizations that require collaborative access to IoT data both within and outside of an organization. The data access is required by various service providers who may be operating as an organization or individual trading businesses. For example, data produced by IoT devices and sensors installed by the smart traffic authority may need to be accessed by their service providers and by other organizations such as smart transports for their operation of smart tram/light rail, etc.

**Figure 1:**
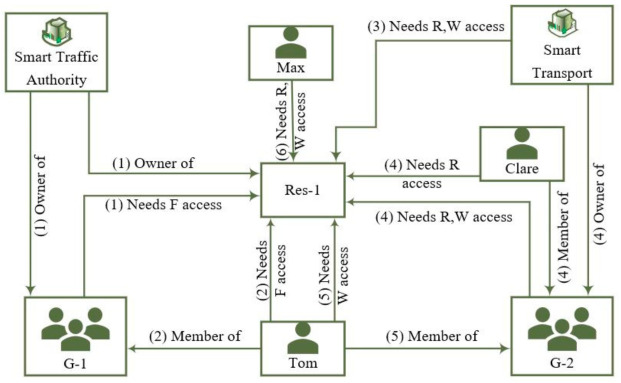
A complex AC use case in a smart city.

An example of a complex access control scenario within a smart city is depicted in Figure 1 where:

1. Smart Traffic Authority Organization has deployed an IoT resource (Res-1). They are the owner of this resource, and its own Group-1 (G-1).
2. Tom is a member of Group-1 (G-1) and needs Full (F) access on Res-1 data.
3. Smart Traffic Authority has agreed to provide Read/Write (R/W) access to the Smart Transport Organization for this resource data.
4. Smart Transport needs to further delegate its R/W access right to its Group-2 (G-2) but wants to further control access to its members Clare and Tom where Clare has a R access, but Tom has a W access.
5. Tom is a casual part-time worker and works for both Smart Traffic Authority and Smart Transport organization.
6. Smart Traffic Authority also agreed to provide a R and W access to an individual third party, Max, who provides some services.

The AC solution must ensure that only legitimate users have access to the data they are officially entitled to. The solution also must support that the latency for data access must be fast (in sub seconds) to be a practical solution that can be adapted in real life.

## 4. Proposed Entitlement-Based Blockchain-Enabled Access Control Architecture

We propose a novel entitlement-based access control architecture that can be enabled using blockchain for any large-scale IoT scenario, which is described below.

### 4.1. System Model for Entitlement-Based Access Control

Entitlement means “having a right to something”. In an IT environment, the term “right” means that a user or a system has the authority to perform an operation. In our proposed entitlement-based access control model, an entitlement is a right granted by a resource owner to a user to perform an operation on the given resource under certain security constraints. The acronyms and definitions used to describe the entitlement model in our proposal are shown in Table 1.

In the proposed system model for entitlement-based AC, access is defined by establishing an explicit Relationship (Rel) between a Party (P) and a Resource (Res) with a set of Operations (Ops) and Constraints (Cs) entitled for the relationship.

It is assumed that within an organizational context, registered Org (e.g., Smart Traffic Authority) is the RO for any Org owned Res and it is also the GO of any G within the Org. Staff users (U) may work within one or more G contexts and a U’s access to an Org Res can be enabled via establishing a GTP relationship between U and Res. Within a G context, a user can be a GM or GA. If any Org (or Ind) wants to provide access to any external P (Org or Ind) for its owned Res, then it can be enabled by establishing a TP relationship (Rel) between the Res and external P. It is also assumed that an Org may want granular control on its Res based on G and U contexts, and a U can have multiple profiles (contexts) for which different access rights may be applied for the same Res.

**Figure 2:**
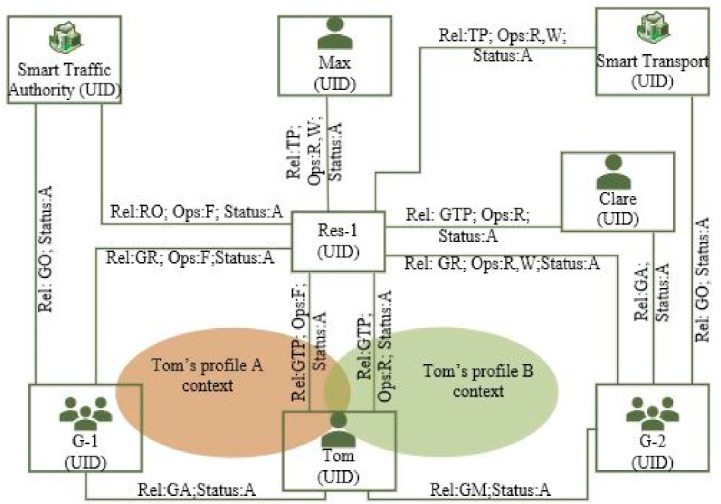
System model for entitlement-based AC for the given smart city use case.

Figure 2 depicts the system model for the proposed entitlement-based AC for the complex smart city use case described in Figure 1. Each party (Org, G, U) and resource is identified by their unique ID (UID). Within the Org boundary, Smart Traffic Authority provides its G-1 an F access to Res-1 by establishing a GR relationship between Smart Traffic Authority and G-1 and then provides user Tom a GA relationship to G1 and a F access by establishing a GTP relationship between Tom and Res-1.

Smart Traffic Authority provides a TP delegated access with R, W for Res-1 to Smart Transport by establishing a TP relationship between Smart Transport and Res-1. Smart Transport now can further delegate its access rights within its own groups and users using the proposed entitlement model. For example, Smart Transport allows G-2 and its users (e.g., Clare has R access vs. Tom has W access) in this example scenario. Smart Traffic Authority also provides a TP access to Max (an Ind entity) to Res-1 with read and write privilege.

**Figure 3:**
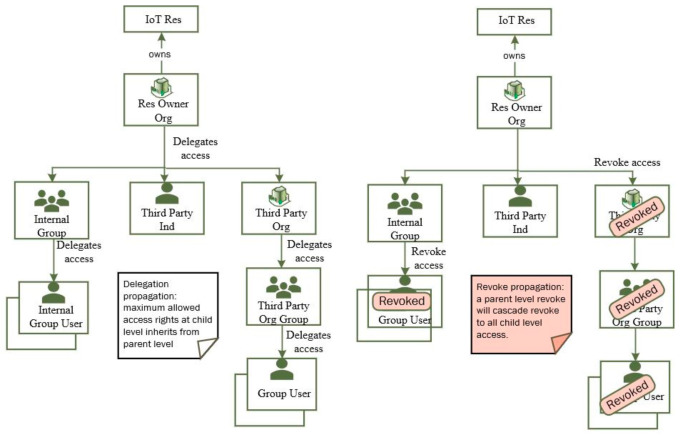
A seamless delegation and revoke propagation in the proposed system model.

As shown in Figure 3, the proposed system model seamlessly supports delegation propagation both within Org and cross-Org scenarios and the highest level of delegated authority is inherited from its parent level. For example, a GR relationship can have lower or same Ops given to its GO. Similarly, U can have lower or same Ops given to its G.

The proposed system model also fully supports access revocation by simply making the relationships status inactive between U and Res. It also supports revoke propagation. Making any parent level relationships (e.g., TP and GR relationships) inactive (invalidate access) will make relationships for all associated U inactive. For example, if Smart Traffic Authority makes TP relationship inactive for Smart Transport, no groups and users within Smart Transport can access Res-1 data anymore.

As shown in Figure 2, the proposed system model also enables users to have context-/profile-based access rights on resources. For example, Tom is a casual member of staff and works in both Smart Traffic Authority and Smart Transport and he has F right within his profile-A vs. W right within his profile-B on the same resource Res-1.

The proposed system model for entitlement-based AC shows that; (1) it provides a scalable AC model and a flexible delegation mechanism for large Org and cross-Org scenarios. The TP Org can also provision fine-grained delegated authority on the given resources using the same entitlement structure, and (2) it also enables a user to execute different access rights based on the user’s profiles.

### 4.2. Proposed Reference Architecture

In this section, we explain our proposed blockchain-enabled access control architecture which uses smart contract to manage entitlements and access control for third parties for IoT resources within smart cities.

**Figure 4:**
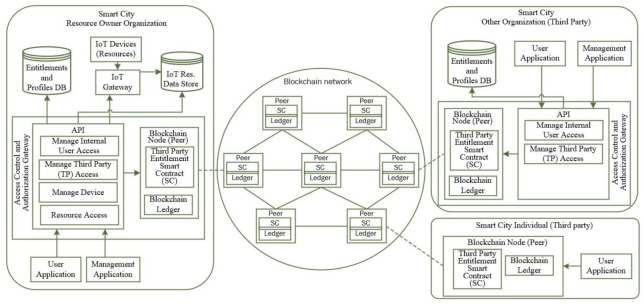
Proposed entitlement-based blockchain-enabled access control architecture for Smart City IoT.

Figure 4 describes the reference architecture of the proposal that can be used for any large-scale IoT scenarios such as a smart city. An earlier version of the architecture appeared in [7].

We propose that either a public or permissioned blockchain with Smart Contract (SC) and entitlements can be used for cross organization access control scenarios while an organization level entitlement using a local database is used for internal access control within single organization scenarios.

**Figure 5:**
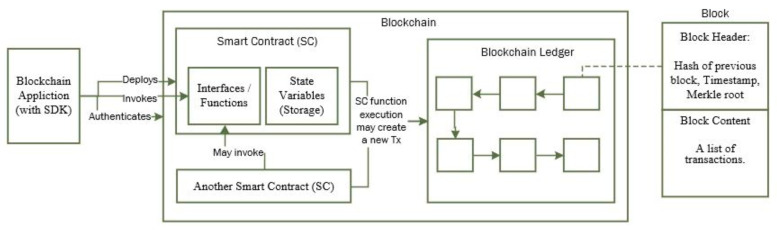
Interaction between application, smart contract and blockchain.

Blockchain systems such as Ethereum and Hyperledger Fabric allow “Smart Contracts” written in procedural language and deployed in blockchain and executed on demand. As with any software program, a smart contract can have its state variables and interfaces (functions) to do something e.g., return the value of any state variables, updating state variables, making call to other smart contract, or creating a blockchain transaction. A smart contract provides tremendous opportunity to build a distributed app (DApp) using blockchain as back-end. The interaction between a blockchain client app with smart contract on blockchain is shown in Figure 5 below. The application needs to authenticate to blockchain using a valid blockchain account before it can deploy a smart contract or invoking a function on smart contract. The application uses blockchain platform-specific SDK (software development kit) for communicating with a blockchain node for all purposes.

Deploying a smart contract (compiled bytecode) requires a blockchain transaction and it creates the storage for all the contract state variables in blockchain and it thus modifies the state of the blockchain. A deployed smart contract in blockchain can be identified with its unique “contract address” which is generated by the blockchain. The client application can subsequently invoke a function on the deployed smart contract. Function within a smart contract can also be called from another smart contract. If the function simply returns the current value(s) of contract variable(s), it does not require a blockchain transaction. However, modifying state variables (residing in blockchain storage) requires a blockchain transaction that needs to be mined and included in the blockchain ledger and will have a significantly longer response time compared to a read function.

The resource owner (RO) organization in our system deploys a smart contract (SC) in blockchain for allowing and managing access to one or many resources to a given third-party (TP) organization. As mentioned earlier, an SC can contain logic and can expose necessary interfaces to interact with it from outside or from within blockchain. In the proposed architecture, SC functions are used to deploy entitlement tokens (containing detailed access right) for third-party organizations and any third-party individuals and generating access tokens that can be presented to resource owner for accessing the given resource. The main components are described below.

Access Control and Authorization Gateway is one (or more) node(s) within resource owner and third-party organizations that joins a blockchain network. The resource owner organization deploys the TP Entitlement Smart Contract in blockchain and subsequently deploys TP entitlement token by making a function call on the SC. The TP organization then can further delegate its access rights and can deploy its user entitlement token by making call to another function on the SC. SC also provides a function for retrieving access token for the TP user that can be used for accessing the resource. This gateway also hosts a list of APIs to support resource owner and TP organization needs and maintains a list of resources and their addresses (access endpoints) that are exposed for external third-party access. APIs include:

- Manage internal user access: the API allows provisioning and maintaining organization’s internal user access and entitlements. This API is used by both resource owner and TP organizations.
- Manage Third-Party (TP) access: This API allows connecting to blockchain and create and manage TP Org entitlements and TP Org user entitlements. This API is used by both resource owner and TP organizations.
- Manage device: this API is used by the resource owner Org for registering the IoT devices.
- Resource access: this API is on the resource owner side and used for accessing the resources. For internal user access for a given resource, the API checks the local entitlement and profile DB before granting resource access. TP user can retrieve the TP user access token from blockchain and make call to this API with the access token and the API validates the token before allowing access.

Entitlements and Profiles DB is a local (off-chain) database that a smart city organization uses to deploy its own user profiles with entitlements to gain access to the org’s own resources.

IoT Devices (Resources) are various resources that generate IoT data. Each resource is identified by a unique identifier.

IoT Gateways aggregate the IoT data and store it in the IoT resource data store. The list of allowed resources that are supposed to store the data is maintained in the IoT gateway.

IoT Resource Data Store stores all the IoT resources data. Depending on the data type and organization’s cloud readiness, it can be either an on-premises store, a cloud, or a combination of both.

User Applications are the applications within an organization that allows users to access organization owned resources and any third-party resources based on a user’s entitlements for a specific context/profile.

Management Application is used by organization staff to register all resources owned by the organization and to manage entitlements for the organization’s internal users and external third-party users. This includes both registering devices and users and provisioning of entitlements and revoking entitlements.

In the next sections, we discuss the detailed design of the entitlement tokens and the smart contract and its interfaces that we have implemented on the Ethereum blockchain platform.

## 5. Entitlement Management and Access Control

This section describes entitlement and access tokens, smart contract interfaces, and the access control flows.

### 5.1. Entitlement and Access Tokens

For third-party cross organization access control scenarios, two types of entitlement tokens are deployed in blockchain within a smart contract (SC). For providing access to a TPGO for one or many resources, the RO organization deploys a smart contract in blockchain and subsequently deploys one or many TPGOEntToken (containing delegated access rights given to a TPGO by the RO for a given resource) by calling a function on the SC. TPGO then can deploy one or many TPGUEntToken (containing delegated access rights given by the TPGO to its users) by calling another function on the SC. The transactions for the TPGOEntToken deployment are digitally signed by the RO and the transactions for TPGUEntTken deployment are digitally signed by TPGO.

The smart contract also provides another function to retrieve an access token for TPGU. The access token generated by smart contract is digitally signed by HmacSha256 algorithm and it uses a secret known by the RO. TPGU Access Token is in the form of Header.Payload.Signature, where Payload is same as the TPGUEntToken, and the signature is the HmacSha256 algorithm output computed on Header + Payload. (HmacSha256 algorithm uses a secret which is known by RO organization only.) Using a lightweight JSON (JavaScript Object Notation) data structure format for the Header and Payload is proposed.

It is assumed that for local resource access within an Org’s internal boundary, relationships between a given resource and user are implemented on the local database.

### 5.2. Smart Contract and Its Interfaces

The resource owner (RO) organization develops and deploys a TP entitlement smart contract (TPEntSC) to provide access rights to an external third-party organization for one or many resources owned by RO. The TPEntSC contract provides the necessary interfaces and data store to manage the above mentioned TPGOEntToken and TPGUEntToken provisioning and generating TPGUAccessToken. The interfaces (functions) on the TPEntSC are shown in Table 2 below. The detailed design of the smart contract and its functions and algorithms are presented in Section 6.

In the proposed architecture, only the TPGO/TPGU entitlements token deployment, subsequent update, and revocation require a blockchain transaction and associated mining operation which typically has higher latency. By contrast, obtaining a TPGUAccessToken from Smart Contract does not require any blockchain transaction as it is a read-only function that does not make any changes in state variables. The latter is much better suited for providing low latency response for large-scale IoT resource access scenarios.

### 5.3. Access Control Flows

1. Entitlement Setup and Revoke for Org’s Users on Local Resources

**Figure 6:**
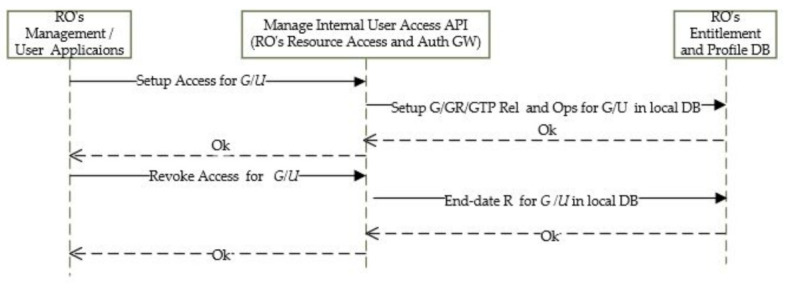
Setup/revoke access for Org’s internal groups ad users.

The entitlements on local resources for an organization’s internal users and groups are set up and revoked via resource owner’s management application, as shown in Figure 6. This starts with the registration of groups and users within a group. The entitlement tokens are stored in local entitlement DB under a specific profile context. To revoke access, the applications will end-date the entitlement in Local DB. End-dating a Rel for a GR will cascade end-dating Rel for all of users for the GR.

2. Entitlement Set-up/Revoke for External TPGO and Their Users

**Figure 7:**
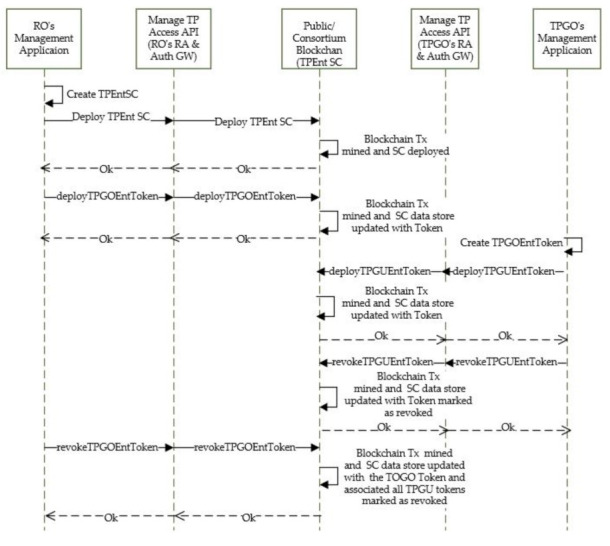
Setup/revoke access for TPGO and their users.

TP entitlements for external group owners are set up by the resource owner’s management application based on agreements between the organizations. As shown in Figure 7, RO’s management application first creates a TPEnt SC and deploys to blockchain. The application then subsequently creates TPGOEntToken (the TP organization level permission on a resource) and deploys it into blockchain by calling the deployTPGOEntToken interface on the SC. TP Organization can then use its management application to create and deploy its user tokens (TPGUEntToken) in blockchain by calling the deployTPGUEntToken interface on the SC. At any time, TPGO can revoke access for any of its users by calling the revokeTPGOEntToken interface on the SC. The revoke call updates the SC datastore by marking the token as revoked. RO also can revoke a TPGOEntToken by making calls to revokeTPGOEntToken interface on the SC. Revocation of the organization level token also does a cascade revoke of all associated TPGUEntToken. In all cases the applications use the Manage TP Access API on the resource access and authorization gateway. As shown in Figure 7, all the token deployment and revoke operations would create blockchain transactions that need to be mined.

3. Device (resource) Registration and Data Aggregation

Resource owner’s management application is used to register all IoT devices in the system and configure the IoT gateway(s) to store the resources data in the IoT datastore. The application uses the Manage Device API on the resource access and authorization gateway. During device registration, the API notifies the IoT gateway to update its eligible list of devices that can store data.

4. Accessing Org Own Resources

Based on the given entitlements for a specific profile context, a user can access the authorized resources via the resource access API on the organization’s access control and authorization gateway. Depending on the privacy needs and sensitivity of the data, the “Resource Access API” can encrypt the data using the requested user’s PK.

5. Accessing External TP Resources

**Figure 8:**
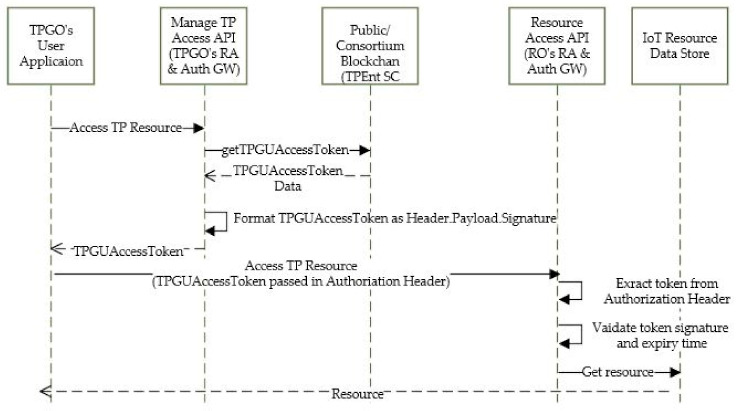
Accessing external TP resources.

Based on the given entitlements for a specific profile context, a TPGU can use the user application to access the authorized TP resources via Manage TP Access API, as shown in Figure 8. The API makes a call to getTPGUAccessToken interface on the smart contract and returns the retrieved TPGUAcessToken to the application. The application reads the token for resource URL (which is behind the RO’s resource access and auth gateway) and makes a call to resource access API on the RO’s site and it passes the TPGUAccessToken in authorization header (as a Bearer token). The resource access API validates the token signature and expiry time before it can return resource data. Again, depending on the privacy needs and sensitivity of the data, the Resource Access API can encrypt the data using the requested user’s PK.

## 6. Smart Contract Detail Design for Ethereum Platform and Algorithms

In this section, we present the smart contract data structures designs and considerations, the algorithms for key interfaces (functions) and theoretical analysis of the time and space complexities for these algorithms.

### 6.1. Data Structure Considerations and Complexities Analysis

Solidity programming language is a popular choice for implementing a smart contract on the Ethereum blockchain platform, and we used solidity for our implementation as well. Solidity supports standard types such as unit, bool, string, bytes32, struct, array plus solidity specific special data types such as address (representing an Ethereum account address) and mapping.

The mapping data structure allows storage as key-value peers and uses syntax-mapping (_KeyType ≥ _ValueType), where _KeyType can be any built-in types plus bytes and strings (but no reference types) and _ValueType can be any type including complex objects and reference types. Implementation of mapping in solidity can be seen as hash tables.

In our implementation, we used struct data structure (e.g., tpgoEntRecord and togoEntRecord) to record a TPGOEntToken and TPGUEntToken. For storing and subsequently searching (look-up) all these records, we considered two alternative data structure designs, such as arrays and mapping data structures in solidity, as shown in Table 3 below. For the mapping option, a byte32 key is generated using a hash of multiple data elements to associate the deployed entitlement tokens, and Ethereum account addresses are used as keys to store the valid RO and TPGO addresses that can have access to the smart contract functions.

For large-scale IoT scenarios such as a smart city, where third-party access needs to be provisioned to many organizations and their users for a huge number of IoT resources, we aim for the key smart contract functions to exhibit a constant time complexity.

**Figure 9:**
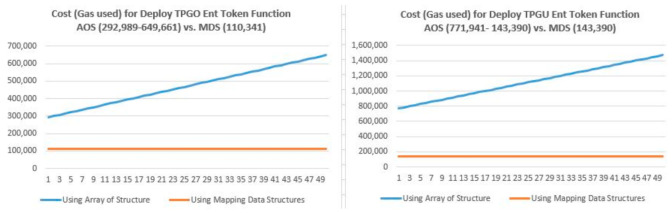
Cost (gas used)—Array of Structure (AOS) vs. Mapping Data Structure (MDS).

A search in an array requires a linear search and thus would add linear complexity for the read and update operations. Since mapping uses hash key and value pairs, a search using mapping types in solidity would add a constant time complexity for the read and update operations. Our experimental results, as presented in Figure 9, for deploying 50 TPGO Entitlement Tokens and subsequently deploying 50 TPGU Entitlement Tokens against two different designs, also show that mapping data structure would lead to significant savings in processing cost (gas used in the Ethereum platform) since it presents constant time complexity vs. linear complexity observed using arrays of structures. Therefore, we used mapping data structures for our implementation in solidity.

Time and space complexity for the smart contract functions using the mapping data structure are shown in Table 4 below. Since mapping data structure implementation uses hash table with Key and value pairs, creating keys and adding to the hash table for deploying a token or an eligible address or searching the table to locate a token and then generating an access token always takes a constant time and hence time complexities of all the smart contact functions is O(1).

Smart contract storage space required to store the tokens and address records increases linearly with the number of records, and hence space complexities for deploying entitlement token functions and adding eligible addresses are O(N). Revoking entitlement tokens, removing eligible address, and Get Access Token do not add any data to smart persistent storage, and hence space complexities for these functions are O(1).

### 6.2. Algorithms for Smart Contract Interfaces

We presented a list of smart contract interfaces (functions) earlier in Table 2. These interfaces are visible to all users of the given Ethereum blockchain network and can make calls to the interfaces. However, we implemented additional security measures in our smart contract interfaces so that the function calls are only allowed from a list of eligible blockchain account addresses for the RO and TPGO organizations. We provided flexibility for both RO and TPGO to maintain the list in the smart contract via its interfaces. More details and algorithms for some of the smart contract interfaces are described below.

1. Smart Contract Constructor

The Smart contract construction function is executed during the smart contract deployment in the blockchain. The objective of the constructor function is to set the necessary variables in the smart contract. In the constructor function, we set the RO and TPGO UIDs and add the initial blockchain account addresses that can be used by RO and TPGO as shown in Algorithm 1. Note that RO and TPGO later can add more addresses by making calls to smart contract interfaces.
**Algorithm 1:** Algorithm to set smart contract variables from constructor function.**Input:** nil **Output:** set the value of some smart contract variables in blockchain 1   roAdrKey ← msg.sender; 2   tpgoAdrKey ← TPGO blockchain account address; 3   roUID ← UID of the RO ; 4   tpgoUID ← UID of the TPGO; 5   roAddresses[roAdrKey] ← 1; 6   tpgoAddresses[tpgoAdrKey] ← 1;

2. Deploy TPGO Entitlement Token

This interface is only allowed to be called from one of the allowed addresses listed in the RO Address list. This interface call is a write/update function and requires a blockchain transaction. If there is already an active TPGO token, the interface assumes that it is an update of the existing token and therefore it revokes all the associated TPGU tokens and then updates the TPGO token value in the blockchain, as shown in Algorithm 2.
**Algorithm 2:** Algorithm to deploy a TPGO entitlement token in blockchain.**Input:** _roUID, _tpgoUID, _resUID, _resUrl, _ops **Output:** TPGO Entitlement Token is added to smart contract storage in blockchain and return success or return error 1   adrKey ← msg.sender; 2   **if** (roAddresses[adrKey] != 1) **then return** error; 3   **if** ((_roUID != roUID) || (_tpgoUID != tpgoUID)) **then return** error; 4   tpgoEntTokenKey ← keccak256 (_roUID, _tpgoUID, _resUID); 5   **if** ((tpgoEntTokens[tpgoEntTokenKey]._isActive) == 1) **then**
6   tpguCount ← (tokenKeys[tpgoEntTokenKey]).length; 7   **if** (tpguCount > 0) **then for each** tpguEntTokens[tokenKeys[tpgoEntTokenKey]]._isActive          ← 0; 8   **end if**
9   tpgoEntTokens[tpgoEntTokenKey] ← tpgoEntRecord (_roUID, _tpgoUID,   _resUID, “TP”, _resURL, _ops, 1); 10   **return** success;

3. Revoke TPGO Entitlement Token

This interface is only allowed to be called from one of the allowed blockchain account addresses stored in the RO addresses list in the smart contract. If the caller address is a valid one, then the interface will revoke the TP entitlement token and will cascade revoke all the TPGU entitlement tokens, if any existing as shown in Algorithm 3. This interface call is a write/update function and therefore requires a blockchain transaction.
**Algorithm 3:** Algorithm to revoke a TPGO entitlement token in blockchain.**Input:** _roUID, _tpgoUID, _resUID **Output:** TPGO Entitlement Token and all associated TPGU Entitlement Tokens are revoked in blockchain and return success or return error 1   adrKey ← msg.sender; 2   **if** (roAddresses[adrKey] != 1) **then return** error; 3   **if** ((_roUID != roUID) || (_tpgoUID != tpgoUID)) **then return** error; 4   tpgoEntTokenKey ← keccak256 (_roUID, _tpgoUID, _resUID); 5   **if** ((tpgoEntTokens[tpgoEntTokenKey]._isActive) == 1) **then**
6   tpguCount ← (tokenKeys[tpgoEntTokenKey]).length; 7   **if** (tpguCount > 0) **then for each** tpguEntTokens[tokenKeys[tpgoEntTo- kenKey]]._isActive ← 0; 8    tpgoEntTokens[tpgoEntTokenKey]._isActive ← 0; 9   **end if**
10   **return** success;

4. Deploy TPGU Entitlement Token

This interface is only allowed to be called from one of the allowed addresses listed in the TPGO address list. This interface call is a write/update function and requires a blockchain transaction. If there is already an active TPGU token, the interface assumes that it is an update of the existing token as shown in Algorithm 4.
**Algorithm 4:** Algorithm to deploy a TPGU entitlement token in blockchain.**Input:** _roUID, _tpgoUID, _tpguUID, _resUID, _tpguPKUrl, _ops **Output:** TPGU Entitlement Token is added to smart contract storage in blockchain and return success or return error 1   adrKey ← msg.sender; 2   **if** (tpgoAddresses[adrKey] != 1) **then return** error; 3   **if** ((_roUID != roUID) || (_tpgoUID != tpgoUID)) **then return** error; 4   tpgoEntTokenKey ← keccak256 (_roUID, _tpgoUID, _resUID); 5    **if** ((tpgoEntTokens[tpgoEntTokenKey]._isActive) != 1) **then return** error; 6   tpguEntTokenKey ← keccak256 (_roUID, _tpgoUID, _tpguUID, _resUID); 7   **if** (_ops > (tpgoEntTokens[tpgoEntTokenKey]._ops)) **then return** error; 8   tpguEntTokens[tpguEntTokenKey] ← tpguEntRecord (_roUID, _tpgoUID,     _tpguUID,_resUID, “GTP”, (tpgoEntTokens[tpgoEntTokenKey]._resUrl),     _tpguPKUrl, _ops, 1); 9   tpguCount ← (tokenKeys[tpgoEntTokenKey]).length; 10   **if** (tpguCount > 0) **then**       **if** any of ((tokenKeys[tpgoEntTokenKey])[ from 0 to (tpguCount-1)] == tpguEntTokenKey) **then return** success; 11   **end If**
12   tokenKeys[tpgoEntTokenKey].push (bytes32(tpguEntTokenKey)); 13   **return** success;

5. Get TPGU Access Token

This interface can be called from any blockchain account owned by TPGO in the network to retrieve a TPGU Access Token. The interface returns the access token Header, Payload, and Signature as shown in Algorithm 5. This interface call is a read-only function and does not require a blockchain transaction.
**Algorithm 5:** Algorithm to create a TPGU Access Token.**Input:** _roUID, _tpgoUID, _tpguUID, _resUID **Output:** returns TPGU Access Token Header, Payload and Signature 1   adrKey ← msg.sender; 2   **if** (tpgoAddresses[adrKey] != 1) **then return** error; 3   tpguEntTokenKey ← keccak256 (_roUID, _tpgoUID, _tpguUID, _resUID); 4   **if** ((tpguEntTokens[tpguEntTokenKey]._isActive) != 1) **then return** error; 5   iat ← block.timestamp; exp ← (iat + token TTL); typ ← “TPGUAccessToken”; alg ← “HmacSha256”; 6   Format Header (in JSON) including iat, exp, typ, alg; 7   Format Payload (in JSON) using data recorded in (tpguEntTokens[tpguEntTokenKey]); 8   Signature ← HmacSha256 ((Header +Payload), Secret); 9   **return** (Header, Payload, Signature);

6. Add or Delete RO Address

This interface is only allowed to be called from one of the allowed addresses listed in the RO Address list. This interface call is a write/update function and requires a blockchain transaction. This interface enables the RO to maintain a list of RO account addresses for this smart contract. Details are provided in Algorithm 6. A similar algorithm is used for “Add or Delete TPGO Address” interface.
**Algorithm 6:** Algorithm to add or remove an eligible RO address.**Input:** _address, _add **Output:** Add (or delete) a RO account address in (from) roAddresses list 1   adrKey ← msg.sender; 2   **if** (roAddresses[adrKey] != 1) **then return** error; 3   **if** ((roAddresses[_address] == 1) && (_add == false)) **then**
roAddresses[_address] ← 0; 4   **else if** ((roAddresses[_address] == 1) && (_add == true)) **then return** error; 5   **else if** ((roAddresses[_address] != 1) && (_add == true)) **then**
roAddresses[_address] ← 1; 6   **else if** ((roAddresses[_address] !== 1) && (_add == false)) **then** return error; 7   **end if**
8   **return** success

## 7. Experimental Results and Evaluations

### 7.1. Experimental Setup

**Figure 10:**
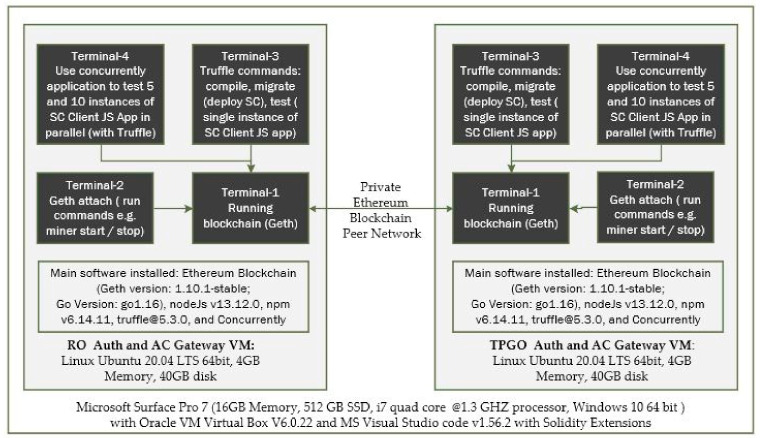
Experimental set-ups for a private Ethereum blockchain network.

Our experimental setup demonstrating a cross-organization scenario is depicted in Figure 10. We created a private Ethereum network using 2× Linux Ubuntu 20.04 LTS (×64) virtual machines deployed on a Microsoft Surface Pro 7 with Window 10 host. The objectives of the experiments are to evaluate performance and scalability of the smart contract functions.

**Figure 11:**
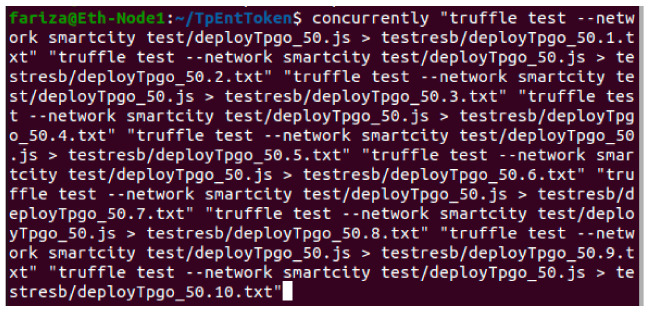
Concurrently-running 10 instances of client Javascript app in parallel.

- We installed the Go version of the open source Ethereum [35] blockchain, Node.Js, NPM, Truffle, and Concurrently applications in each virtual machine and created a private blockchain network.
- We used multiple terminal windows to run the blockchain nodes and test smart construct deployment and its interfaces. From Terminal-1 we run Geth commands to initialize and start the blockchain. In Terminal-2, we run Geth attach command to connect to the running instance of blockchain and run the control commands such as start/stop miner and peering the nodes.
- Truffle is used in Terminal-3 to compile and deploy smart contract bytecodes to the blockchain network. Truffle is also used to run a single instance of our Javascript client application that makes single call or make repeated calls (e.g., making a consecutive 10,000 calls to Get TPGU Access Token interface, making a consecutive 500 calls to Deploy TPGU Entitlement Token etc.).
- In order to simulate and test the TP access control for a large IoT network such as smart city, we used the Concurrently application (in Terminal-4) to instantiate parallel instances (×5 and ×10) of our Javascript client application so that we can test the scalability of both deploy entitlement token functions (updating blockchain) and obtain the access token function (reading from blockchain and generating access token). We tested 10,000 read calls and 500 writes/updated calls simultaneously running from 5 and 10 instances of client application concurrently. An example of the concurrently command is shown in Figure 11.

### 7.2. Experimental Results

1. Testing Smart Contract Deployment

**Figure 12:**
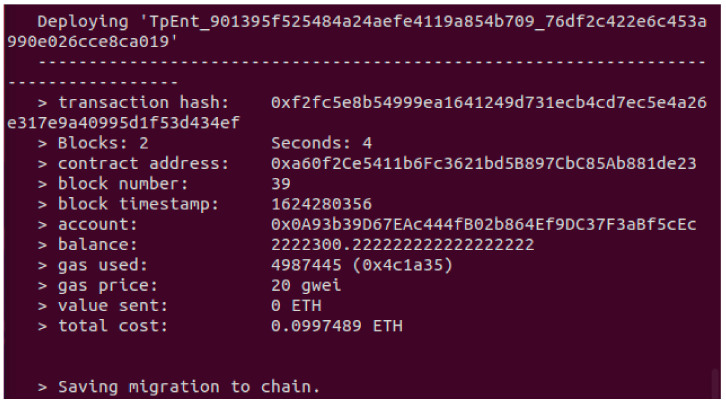
Contract deployment test results from Truffle Command Terminal and Geth console.

Contract deployment in a private blockchain network took 1–4 s. The result from a deployment test is shown in Figure 12 below.

2. Testing Smart Contract Interfaces—Integration Testing

**Figure 13:**
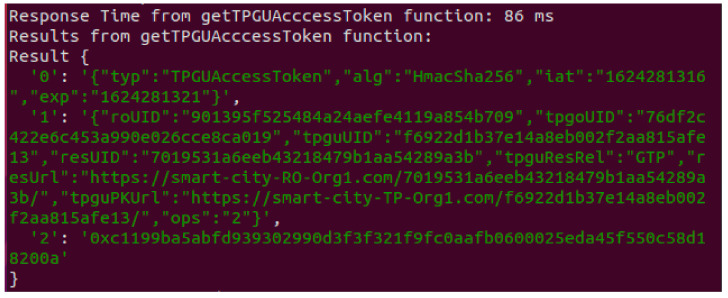
Test result from Get TPGU Access Token function call.

We developed JavaScript scripts (application) to test all the interfaces of our smart contract. We used Terminal-3 and have tested our scripts using Truffle test utility for both success and various failure scenarios to ensure that the smart contract functions are working as per the design and as expected. Response from a Get TPGU Access Token (Read Function) call is shown in Figure 13. Table 5 shows the response (time and part of transaction receipts) from a call to all other smart contract interfaces (write/update functions) with response times varying from 3210 ms to 5225 ms.

3. Testing Smart Contract Interfaces—Performance

**Figure 14:**
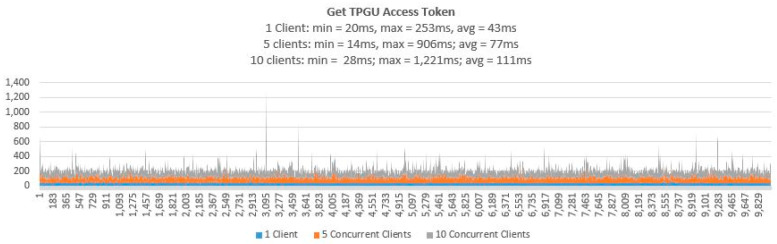
Response times from Get TPGU Access Token for 10,000 calls.

**Figure 15:**
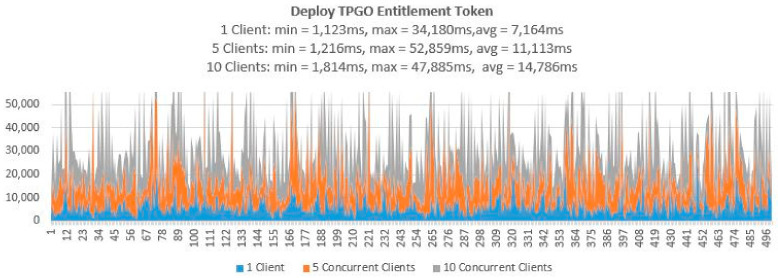
Response times from Deploy TPGO Entitlement Token for 500 calls.

**Figure 16:**
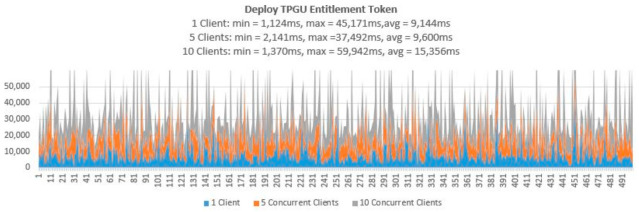
Response times from Deploy TPGU Entitlement Tokens for 500 calls.

**Figure 17:**
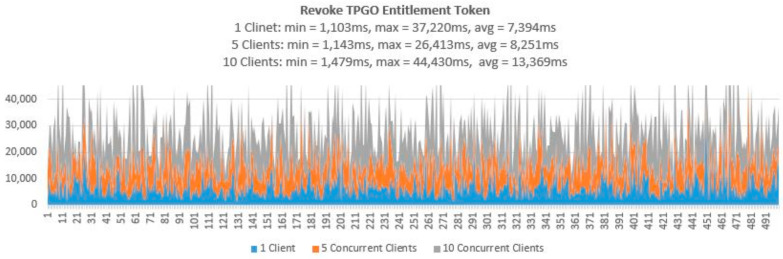
Response times from Revoke TPGO Entitlement Token for 500 calls.

**Figure 18:**
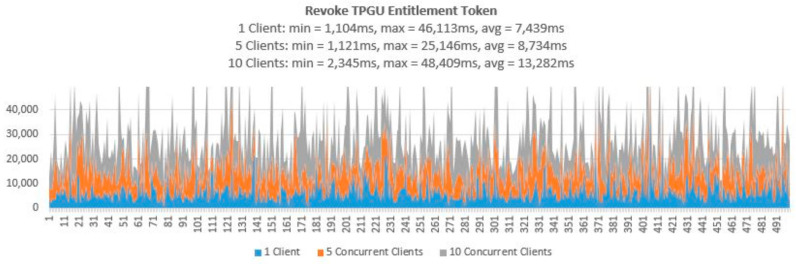
Response times from Revoke TPGU Entitlement Token for 500 calls.

To demonstrate that the proposed access control solution using smart contract and blockchain can be used for large IoT scenarios such as smart cities, we performed extensive testing for Get TPGU Access Token functions and the Entitlement Token deployment and revoke functions. Each function was tested using 3 load testing set-ups as presented in Table 6 and the response times from the tests are shown in Figure 14, Figure 15, Figure 16, Figure 17 and Figure 18.

**Figure 19:**
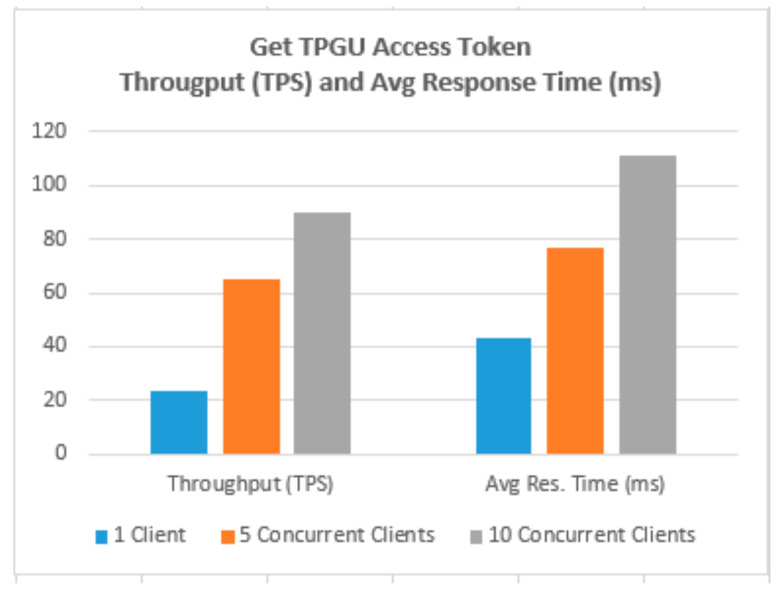
Throughput (TPS) vs. Average Response Time (ms) for Get Access Token Function.

**Figure 20:**
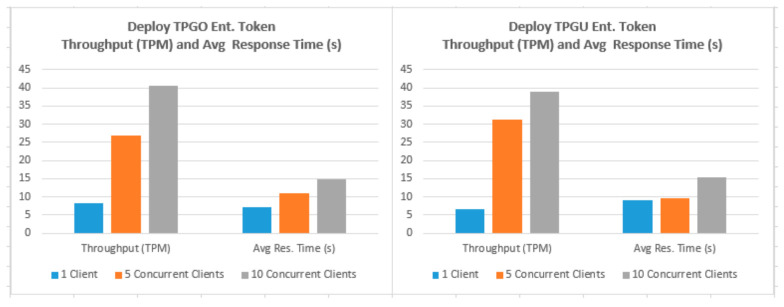
Throughput (TPM) vs. Average Response Time (s) for Deploy Token Functions.

**Figure 21:**
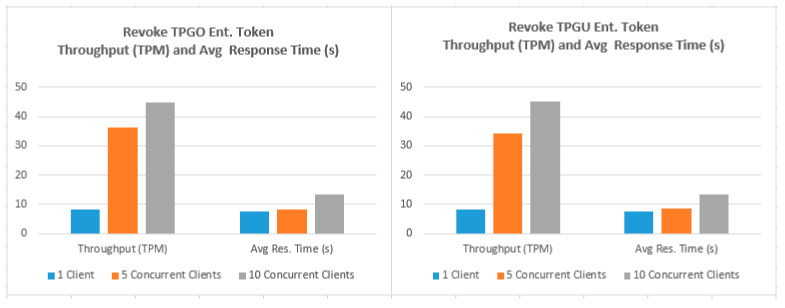
Throughput (TPM) vs. Average Response Time (s) for Revoke Token Functions.

We also measured the throughput for TPGU Access Token in terms of Transactions (Calls) Per Second (TPS) and throughput for tokens deployment and revoke interfaces in terms of Transactions Per Minute (TPM) and they are presented against the average response time for all load testing scenarios in Figure 19, Figure 20 and Figure 21. Analysis of the results is presented in Section 5.3.

It may be noted that the add/delete RO or TPGO address functions are not volumes tested because we do not expect the usage of these two functions to be high enough.

### 7.3. Experimental Results and Evaluation

For the large-scale IoT access control scenarios, response times for retrieval of the access token are crucial from the perspectives of scalability and performance. Therefore, we carried out volume testing of 10, 000 calls for Get TPGU Access Token interface with three load testing setups with one client, five concurrent clients, and 10 concurrent clients generating load, and average response time measured (as shown in Figure 14) were 43 ms, 77 ms, and 111 ms, respectively, in our private Ethereum network.

All the smart contract functions that change the state variables and the data in smart contracts take naturally longer times since they generate blockchain transactions that need to be mined first before Ethereum can make a call back to the caller with transaction receipts. As shown in Table 5, integration testing in our private Ethereum blockchain making single calls for different write/update functions took 3210–5225 ms. As shown in Figure 15, Figure 16, Figure 17 and Figure 18, average response times from volume testing of 500 calls for deploy and revoke entitlement token interfaces varied from 7163 ms to 15,356 ms, depending on load testing set-ups. The minimum response times for all the token deployment and revoke functions were under 2000 ms. We observed occasional spikes of higher response times for the token deployment and revoke functions for all load testing scenarios with maximum response times for token deployment functions were higher than 47,000 ms and maximum response times for token revoke functions were higher than 25,000 ms.

Transaction throughput against average response times for all load testing scenarios for the given functions, as presented in Figure 19, Figure 20 and Figure 21, provides some interesting findings. The throughput in terms of transactions per second (TPS)/transactions per minute (TPM) improves significantly (e.g., increased by 179–348%) from one client load testing scenario to five clients load testing scenarios without significant degradation of average response times (e.g., response times increased by 5–79%). By increasing the concurrent clients from 5 to 10, the throughput only increased by 23–50% with average response time increased by 33–62%.

As shown in Figure 19, Get TPGU Access Token function achieved TPS throughput of 23–90 TPS, which is equivalent of 662,400–2,592,000 Get Access Token function calls per day (@ 8 h a day) with an average response time of 43–111 ms. The throughput and the response time for the access token generation and retrieval shows that the smart contract in Ethereum platform can meet the demand of a large-scale cross-organizational access control scenarios such as smart cities without any issues. We argue that the TPS for Get Access Token function should be well below 20 TPS for a cross organization IoT access control scenario and hence the response time for the access token retrieval to be below 50ms for our scheme.

As shown in Figure 20, the throughput for deploy token functions varied from 7–40 TPM (depending on load testing scenarios), which is equivalent to provisioning 3360–19,200 new entitlements token per day (@ 8 h a day). As shown in Figure 21, the throughput for revoking token functions varied from 8–45 TPM (depending on load testing scenarios), which is equivalent to revoking 3840–21,600 tokens per day (@ 8 h a day). We argue that the average response times (e.g., 7.164–15.356 s) tested for token deployment and revoke functions are acceptable since the token deployment (provisioning) and revoke functions are supposed to be management functions that are to be carried out one-off or in-frequently (compared to Get Access Token function).

It may be noted that a transaction (i.e., an update/write function call) in Ethereum public network may need 10–15 s. If the implementation is carried out in a permissioned network, the transaction time would be reduced. It may be emphasized that for all blockchain networks scenarios (public or permissioned), our Get TPGU Access Token function, which is essentially providing third-party user access authorization and providing access token, would not need any blockchain transactions and hence response time would be in the lower order of milliseconds (i.e., below 50 ms).

To compare our results with relevant literatures presented in Section 2 earlier, Zhang et al. [6] and Sultana et al. [12] Ouaddah et al. [13] proposed some blockchain-based access control framework for IoT, but their proposed schemes require a response times in the order of seconds for access authorizations (compared to our <50 ms response times) since their access authorization is fully dependent on blockchain transactions that requires to be mined before access authorizations can be granted.

Our experimental results presented above prove that the proposed entitlement-based blockchain- and smart contract-enabled IoT access control architecture can be implemented and be used in real world large-scale IoT environments.

### 7.4. Evaluation of Security and Privacy

The proposed security architecture addresses all the key IoT security and privacy concerns. A qualitative evaluation is presented in Table 7.

## 8. Conclusions

The proposed novel entitlement-based access control model provides a scalable and flexible access control mechanism that supports low-latency for cross-organization scenarios that utilize large-scale IoT networks. Our proposal provides a flexible delegation mechanism which supports propagation and inheritance, and the third party can further delegate its access rights to its users and groups without resource owner losing any control. Our model also provides a robust revoke mechanism with revoke propagation within organization and groups. The architecture allows the smart contract to be deployed either in a public or permissioned blockchain network.

The experimental evaluation using private Ethereum blockchain and smart contracts with extensive performance testing with concurrent clients (load generator) proves that the third-party entitlement tokens can be easily coded in a smart contract and deployed in the blockchain. Subsequently, the blockchain can be used for access authorizations and generating digitally signed access tokens for IoT resource access. The throughput and response times from the access token retrieval, entitlement token deployment and revoke, proves that the architecture is scalable and can be used by any large-scale IoT applications including smart cities. We are planning to implement the proposed solution in a large-scale test Ethereum network as our future work. We also aim to implement our proposed solution for another IoT use case scenario such as smart agriculture.

## Figures and Tables

**Table 1:** Acronyms and Definitions.

Acronym	Description
Org	An organization entity or domain. It is the registered legal business entity that runs the organization.
Ind	An individual entity.
Res	An IoT Resource.
RO	Resource Owner. It could be an Org or Ind entity.
G	A Group (division) within an Org.
GO	Group Owner. It could be either RO Org or TP Org.
P	A party. It is an entity representing an Org, Ind, G, or U.
GM	A Group Member. GM represents a relationship between an Ind and G.
GA	A Group Admin. GA also represents a relationship between an Ind and G. (GA is a GM with admin privilege).
U	A User. It is an Ind.
TP	Third Party. An external Org or external Ind.
TPGO	Third-Party Group Owner.
TPGU	Third-Party Group User.
GTP	Group Third Party. A party within an Org G.
GR	Group Resource. It is an RS made available for a G.
Rel	Relationship between a P and RS or between Ps (parties). In the proposed model, Rel can be labelled as RO, GO, GR, TP, GTP, GM, or GA.
Ops	A set of Operations (e.g., Read (R), Write (W), F (Full)).
Cs	A set of constraints (e.g., Status (Active(A)/Inactive(I))).

**Table 2:** Third-Party Entitlement Smart Contract Interfaces (Functions).

Interface Name	Description
deployTPGOEntToken	This interface is called by the RO to deploy a TPGOEntToken.
deployTPGUEntToken	This interface is called by the TPGO to deploy a TPGUEntToken.
getTPGUAccessToken	This interface is be called by the TPGO network to retrieve the TPGUAccessToken (which is presented to the resource owner to gain access on the given resource).
revokeTPGUEntToken	This interface is called by the TPGO for revoking a TPGUEntToken.
revokeTPGOEntToken	This interface is called by the RO to revoke the TPGO token. The interface also carries out a cascade revoke for all the associated TPGUEntToken.
addDeleteTPGOAddress	This interface can be called by the TPGO for maintaining a list of valid blockchain account addresses for TPGO for accessing the smart contract.
addDeleteROAddress	This interface is called by the RO for maintaining a list of valid blockchain account addresses for RO for accessing the smart contract.

**Table 3:** Data storage alternative considerations.

Data Storage—Using Array	Data Storage—Using Mapping
tpgoEntRecord[] private tpgoEntTokens;	mapping (bytes32 ≥ tpgoEntRecord) private tpgoEntTokens;
tpguEntRecord[] private tpguEntTokens;	mapping (bytes32 ≥ tpguEntRecord) private tpguEntTokens;
addressRecord[] private tpgoAddresses;	mapping (address ≥ uint) private tpgoValidAddresses;
addressRecord[] private roAddresses;	mapping (address ≥ uint) private roValidAddresses;

**Table 4:** Time and space complexity for smart contract functions using mapping data structure.

Functions	Deploy Entitlement Tokens, Add Eligible Addresses	Revoke Entitlement Tokens, Remove Eligible Addresses	Get Access Token
Time	O(1)	O(1)	O(1)
Space	O(N)	O(1)	O(1)

**Table 5:** Response Times and TX receipts (partial) for update interfaces in SIT.

Interface	Test Results with Response Time and Transaction Receipt (Partial)
Deploy TPGO Ent Token	Response Time: 3210 ms; transactionHash: 0xed5412899c4129547bcd07745cec2e2bbbfb1250f3c17647aa8d1e4ab96f487;blockHash: 0xad09c089c20423c44733951a5d0a54dc92140595ca9e00a8ab5d50180c3ce95; gasUsed: 284945
Revoke TPGO Ent Token	Response Time: 4172 ms; transactionHash: 0 × 4fd852805a1a33aeb97e9b50438c0d5e21e58c24a509cda919d7969dbce06ba4;blockHash: 0xd0884ca4db333629671ddb4bfc5d21c00bec704e0fb913b96bd46fa49d6943; gasUsed: 26,293
Deploy TPGU Ent Token	Response Time: 4199 ms; transactionHash: 0xffd3f29cc74fd0e75eb49bb3b431ec2b1c9de769e0d5c9ab470f9f4873484347;blockHash: 0xa696ae2f8c7ca162f37bc7e4ba0b3537f6ed90246df04a7d6c89c2dffbf5505c; gasUsed: 433,140
Revoke TPGU Ent Token	Response Time: 4212 ms; transactionHash: 0x43b0a1c6ccac6d2d3d6bfc649dbb6414e3247c7cff27bae639ca04c95d38135;blockHash: 0x160dbc93126c99effb103ac32f6403aaabc8261480f56143928604de50773dc; gasUsed: 29,271
Add or Delete RO Address	Response Time: 5225 ms; transactionHash: 0x02cf9483a40a58ab2a5bf4e0158e144e8fc00a6c26d17874bc1b95ed187de4fa; blockHash: 0x7c664750eaf0f2c9e737e5e207d4e790eed70d2200b3af36a4bd5ad98eed79c; gasUsed: 44,485
Add or Delete TPGO Address	Response Time: 4263 ms; transactionHash: 0x3f9c5f2870d8cff7765e4fe121d52699bce86f25575a47d3e5c46bf7b5e08e56; blockHash: 0xd2b8681c8b24bd2f4b8fc023939289df12da44c4e49131fbc06d5133789400c; gasUsed: 44,484

**Table 6:** Performance Testing Set-ups.

Interface	Total Volume (# Calls)	One Client (# Calls)	Five Concurrent Clients (Each # Calls)	Ten Concurrent Clients (Each # Calls)
Get TPGU Access Token	10,000	10,000	2000	1000
Deploy TPGO Ent Token	500	500	100	50
Revoke TPGO Ent Token	500	500	100	50
Deploy TPGU Ent Token	500	500	100	50
Revoke TPGU Ent Token	500	500	100	50

**Table 7:** Qualitative Evaluation of Security and Privacy Concerns.

Concerns	Definition	How It Is Addressed in The Proposed Architecture
Authentication	Authentication verifies user identity.	Organization-specific Authentication (Auth) mechanism will apply for resource owner and user applications. BC nodes will use their BC accounts. Standard Auth using functional logon, mutual Auth, API keys etc. to be used for system-system access.
Access Control	Access control ensures legitimacy of user’s access on a resource.	Entitlement-based and blockchain-enabled access control for TP access as presented in the paper above. Entitlement-based access control for organization’s internal users. The proposed architecture also provides robust revoke mechanisms with revoke propagation. Smart contract functions are designed utilizing “Secure by Design” principles. For instance, each of the function does the validations and reject the calls if the callers are not authorized and/or the invalid data are passed in. The design also allows flexibility of maintaining a RO and TPGO account address list by the legitimate users. If an account becomes compromised, the RO and TPGO can delete (i.e., make invalid) an address.
Privacy/Anonymity	User’s identity is not identifiable to public.	No personally identifiable information (PII) data are deployed in BC. SSL tunnel encryption applies for all communications. Access token will be included in authorization (bearer) header which will be SSL tunnel encrypted. Additionally, access token will be digitally signed (using HmacSha256 algorithm using a secret known by the RO).
Confidentiality	Information for a user is made unintelligible to others	In the proposed solution, user’s public key URL will be part of user’s access token and the data can be encrypted with the user’s public key by the resource access gateway before returning the data.
Integrity	Ensuring that data is not modified by un-authorized party.	Standard-based process and mechanisms will be applied for data at rest by the RO. Any tampering of entitlement tokens in BC is protected by validating that the caller’s blockchain account address is eligible. Additionally, any tampering of the access token is protected by digital signature.
Availability	Ensuring that services are available to legitimate users.	It is assumed that industry standard-based policies and security controls (including firewalls/appliances) will be used by each organization. Moreover, both gateways will validate the access rights to eliminate any Denial of Services (DoS) attacks.
